# Artificial Intelligence: A New Paradigm in Obstetrics and Gynecology Research and Clinical Practice

**DOI:** 10.7759/cureus.7124

**Published:** 2020-02-28

**Authors:** Pulwasha Iftikhar, Marcela V Kuijpers, Azadeh Khayyat, Aqsa Iftikhar, Maribel DeGouvia De Sa

**Affiliations:** 1 Obstetrics and Gynecology, St. John's University, New York, USA; 2 Obstetrics and Gynecology, Universidad de Ciencias Medicas, San José, CRI; 3 Internal Medicine, Ahvaz Jundishapur University of Medical Sciences, Ahvaz, IRN; 4 Bioinformatics, City College of New York, New York, USA; 5 Obstetrics and Gynecology, Aberdeen Royal Infirmary, Aberdeen, GBR

**Keywords:** artificial intelligence, obstetrics, gynecology, cost effectiveness, machine learning, pregnancy surveillance, preterm labor, fetal heart rate, in vitro fertilization, cancer screening

## Abstract

Artificial intelligence (AI) is growing exponentially in various fields, including medicine. This paper reviews the pertinent aspects of AI in obstetrics and gynecology (OB/GYN) and how these can be applied to improve patient outcomes and reduce the healthcare costs and workload for clinicians.

Herein, we will address current AI uses in OB/GYN, and the use of AI as a tool to interpret fetal heart rate (FHR) and cardiotocography (CTG) to aid in the detection of preterm labor, pregnancy complications, and review discrepancies in its interpretation between clinicians to reduce maternal and infant morbidity and mortality. AI systems can be used as tools to create algorithms identifying asymptomatic women with short cervical length who are at risk of preterm birth. Additionally, the benefits of using the vast data capacity of AI storage can assist in determining the risk factors for preterm labor using multiomics and extensive genomic data. In the field of gynecological surgery, the use of augmented reality helps surgeons detect vital structures, thus decreasing complications, reducing operative time, and helping surgeons in training to practice in a realistic setting. Using three-dimensional (3D) printers can provide materials that mimic real tissues and also helps trainees to practice on a realistic model. Furthermore, 3D imaging allows better depth perception than its two-dimensional (2D) counterpart, allowing the surgeon to create preoperative plans according to tissue depth and dimensions. Although AI has some limitations, this new technology can improve the prognosis and management of patients, reduce healthcare costs, and help OB/GYN practitioners to reduce their workload and increase their efficiency and accuracy by incorporating AI systems into their daily practice.

AI has the potential to guide practitioners in decision-making, reaching a diagnosis, and improving case management. It can reduce healthcare costs by decreasing medical errors and providing more dependable predictions. AI systems can accurately provide information on the large array of patients in clinical settings, although more robust data is required.

## Introduction and background

Artificial intelligence (AI) is a type of digital computer system that parallels the way the human brain processes information. AI is organized in a similar way that neurons in the brain are arranged, with their multiple neural nodes, and so are referred to as neural networks [1-2]. The rise of AI has led to the subsequent development of artificial neural networks (ANN), which consist of a dependable mathematical system that can interpret multifactorial data [3-5]. These neurons are connected via multiple synapses and send the data to each other back and forth, and by doing so, come up with the most probable answer. Making these multiple connections enables computers to mimic cognitive functions, such as the reasoning process, to identify the most probable answer to a problem. This complex algorithm AI software is now utilized in medicine to analyze large amounts of data, which can assist in disease prevention, diagnosing, and monitoring patients [2]. In recent years, there has been an enormous rise in the global growth of artificial intelligence in healthcare systems. According to statistics, expenditure on AI is expected to increase from $2.1 billion to $36.1 billion by 2025 [3]. Artificial intelligence requires training and collaboration between partners for it to become a success in healthcare. One example is IBM's Watson for Oncology (IBM Corp., Armonk, NY), a well-known AI technology that has received appraisal from customers, which is mostly used for prescription information extraction from various drug databases and medical journals [4].

Overall, AI can aid practitioners in decision-making and will help clinicians to make more self-assured decisions. However, it is important to keep in mind that it is not a substitute for clinical experience [3]. The third leading cause of death in the United States (US) is medical errors, and AI can potentially help reduce them by improving accuracy in interpretation and decreasing the workload that can lead to details being overlooked [5]. In this article, we introduce different types of AI, their potential applications, associated research, benefits, limitations, and the future of AI, as well as how it can benefit the field of obstetrics and gynecology (OB/GYN).

## Review

Fetal heart monitoring and pregnancy surveillance

Fetal heart rate (FHR) monitoring helps the healthcare provider to monitor the fetus and diagnose associated high-risk complications. It also gives a qualitative and quantitative overview of baseline FHR, variability, acceleration, deceleration, uterine contraction intensity, and FHR pattern changes [6]. Currently, AI is used to monitor the FHR rate during labor via analyzing cardiotocographs and estimating possible outcomes. As Desai stated in his study, the benefits of its incorporation in obstetrics, specifically antepartum monitoring, would be advantageous [2]. This technology would help to decrease the discrepancies between different obstetricians interpreting intrapartum monitoring, thus providing a more reliable and replicable output for each analysis, and ultimately reducing perinatal and maternal complications and morbidity. Artificial intelligence systems can also provide supporting evidence in cases of unpredictable poor outcomes that can potentially result in litigation. One of the examples of where AI was tested in cardiotocography (CTG) analysis was published in 2018 where the CAFE (Computer-Aided Fetal Evaluator) studied the possibility of an AI system being able to interpret CTG data [2]. The results concluded that the AI system read the information at a similar level as the experts in the field and was also able to detect errors. Some limitations in this study were disagreements between specialists when interpreting some data, mostly in interpreting variations, which led to an inability to conclude if the error in interpretation arose from a problem in the system or in obtaining agreement between experts in the field [7]. This was an obstacle in proving that the system is optimum and confirming its precision. More robust research is required to enhance flexibility in data interpretation.

CTG technology arose 50 years ago, and frequent differences between specialists urge for a system that decreases error and unifies interpretation while analyzing CTG. The Computerised Interpretation of Fetal Heart Rate During Labour (INFANT) study protocol is a large trial currently evaluating the ability of AI interpretation of CTG during labor to assist practitioners in deciding the best management on an individual basis [8]. The goal is to make FHR reading more reliable, to aid the physician in interpreting and decision-making, and decrease the burden of work by making it more efficient. Perinatal asphyxia is a significant problem worldwide, and by creating an efficient way to monitor FHR, it would improve care and decrease poor outcomes. Thus, an alarm monitor that warns of potential fetal distress can guide the practitioner to act in a timely matter and perform intrapartum interventions when necessary. It can also provide reassurance and avoid unnecessary treatment in those women where fetal distress is not observed.

System 8000 is a computer system that analyzes the antenatal FHR [8-9]. It monitors changes in FHR and detects amplitudes associated with hypoxemia by detecting decelerations and changes in variability. Currently, a decrease in variation is the most dependable index of fetal deterioration, but unfortunately, there is significant observer variation in interpreting this data. The introduction of AI can decrease this difference between readers, which can lead to a more reliable interpretation of variation, decrease unnecessary interventions, and expedite the delivery if necessary.

A study was done by Kazantsev et al. urges the development of new technologies that can address the issue of maternal and infant mortality [10]. The researchers in this study believed that AI technology could be used for outpatient care in the form of home monitors that can adequately provide surveillance of high-risk patients. This technology, in conjunction with telemedicine, can potentially aid in earlier detection of potential complications and reassure the clinician and the patient that a safe system of monitoring is being used, even if at an outside clinic or hospital care. It also provides a warning system that informs the patient of dangerous FHR readings and signals them to notify the physician to decide on further care according to the in-home AI analysis. In this study, they used neural network recognition algorithms that could provide a solution to interpretation discrepancies between specialists. It also mentioned that outpatient monitoring is readily accessible because most homes have access to the Internet and smartphones. The insertion of AI into Doppler ultrasound has proven to be cost-effective. The system was also able to exclude readings of uncertain meaning, such as pseudo accelerations and pseudo decelerations that occur due to intense fetal movement, thus not falsely alarming the patient of possible fetal endangerment. The downside of the study was that the proposed method was only tested in one subject, and even though it proved successful, a larger study is needed to replicate its results and validate this intervention. The possibility of guiding decision-making and management using telecommunications, combined with in-home pregnancy monitoring, can prove beneficial in the early detection of pregnancy complications and decrease maternal and infant mortality [8-10].

Gestational diabetes mellitus

Current screening for gestational diabetes mellitus (GDM) is costly and a burden for pregnant women. AI can assist in finding ways to replace the current standard. Current screening guidelines, according to the United States Preventive Services Task Force, consist of a 50 g of oral glucose challenge test performed after 24-week gestation, followed by a second diagnostic test. They found insufficient evidence to screen before 24-week gestation [11]. Screening for GDM is important to prevent complications, such as hypoglycemia in the neonate, large for gestational age, and an increased number of cesarean births, amongst others. GDM also results in more expensive outcomes due to increased cesarean deliveries, which could be decreased if a more convenient screening test was available. Polak and Mendyk created a study to evaluate the use of an AI calculator to screen for GDM that would be more cost-efficient and less inconvenient for the patient than current guidelines [12]. They proposed the use of an online calculator that a physician and patient can use for screening. The calculator was based on an artificial neuronal network (ANN) which was adapted to a Java applet and published online [12]. The model they used to create the AI system is called multi-layer perceptrons, which can send information back and forth, hence its denomination as an algorithm-back propagation (BP). Risk factors, such as high blood pressure, hyperlipidemia, smoking, weight, low-fat diet, and ethnicity, are used to create the calculator. The authors concluded that despite AI having lower-efficacy than the standard screening test, the current ANN model on the website will continue to progress and learn as it continues to be exposed to more cases, with the finality of eventually helping to lower health costs [13]. An additional benefit is that it is user-friendly, which makes it practical for daily use.

Preterm labor

Innovative research by Singh et al. studied the combination of AI and amniotic fluid (AF) proteomics and metabolomics, in conjunction or independently with imaging, demographic, and clinical factors, to predict perinatal outcomes in asymptomatic women with short cervix length [14]. The type of AI they used was called deep learning (DL). This subtype of AI can operate with a larger amount of data because it has a greater number of neural networks, which makes it ideal for biological system studies involving multiomics. Currently, the short cervical length is the strongest risk factor for prematurity; however, many women with this condition carry their pregnancy to term. Many centers now incorporate amniocentesis in these women to evaluate additional factors that might put them at risk, such as inflammation and infectious processes. The AF of the subjects was additionally studied for omics, such as metabolomics, to shed light on potential new biomarkers that might be involved in preterm birth. This can improve the accuracy and predictive value of women at risk of poor outcomes. It can also help physicians stratify those patients at risk of preterm birth better than the current risk factors, such as short cervical length and prior preterm birth delivery [15]. In this way, physicians can use this tool to guide their management, such as observation alone, or suggesting cervical cerclage and or antenatal steroids if deemed necessary. A shortcoming of the study was the small size of the group. The study concluded that DL was a superior tool when it came to the prediction of perinatal outcome in asymptomatic women with short cervix length and that further studies are needed to analyze AF omics and its relationship to premature shortening of the cervix to help guide management in these patients.

A study done by Idowu et al. emphasized the importance of using AI technology to decrease expenses generated by inaccurate detection of preterm labor leading to unnecessary hospitalizations and procedures, and in the meantime, expedite treatment in those who are in true labor to prevent hazardous consequences for the baby and the mother [16]. In this study, they used electrohysterography (EHG) signals and used three distinct machine learning algorithms to classify these signals to help them identify true labor and accurately diagnose preterm labor. They concluded that the random forest algorithm performed the most efficiently of the three machines tested, as it was able to handle a larger amount of data, was relatively accurate, and had a robust learning capacity which resulted in an accuracy of 97% in predicting preterm labor.

Parturition

The onset of labor can lead to complications when this occurs before or after the ideal time frame, such as preterm or post-term pregnancy. To get a better understanding, Mason et al. used gene array profiling of myometrial events during guinea pig pregnancy to achieve a better comprehension of the molecular mechanisms that regulate labor [17]. In this study, they used AI technology to develop diagrams composed of gene circuits which helped them in extracting the pertinent information about myometrial activation from a considerable amount of data. However, this study was done in the myometrium of guinea pigs, and there is yet to be a study illustrating this in humans. Norwitz described AI use to help in solving issues related to parturition but recommended further studies be done to understand the genes involved in human parturition and validate these results [18]. Additionally, we need to consider variables, such as the complications of pregnancy, and how these factors can alter myometrial gene expression. This study gave an overview in our understanding of how genes that are activated during labor can potentially lead to effective medical interventions when necessary, thus helping to treat some of the disorders of parturition, such as preterm labor and post-term pregnancy, and decrease the associated perinatal morbidity and mortality involved with these complications. 

An example of a novel program that can aid with the task of understanding how gene expression in the myometrium is programmed is the MetaCore program [19]. It consists of a knowledge database and software that can analyze data and gene lists. Its limitations are that it needs prior knowledge about the problem or a predefined algorithm. Reasons for preterm or post-term labor are multifactorial, and not all factors are known or well understood [17-19]. Nonetheless, other programs that do not require prior knowledge or utilize a defined algorithm, such as neural networks, can overcome these aforementioned limitations. MetaCore is also able to analyze large amounts of data, such as genomes and their variables, and analyze it nonlinearly. A disadvantage is that it requires a significant amount of time and research because all simulated computer predictions need to be tested in humans to confirm their accuracy and practicability.

In vitro fertilization

A study done by Guh et al. used data mining and AI to create a computer algorithm that can help predict pregnancy using in vitro fertilization (IVF) [20]. Data mining (DM) uses AI in conjunction with advanced statistics to discover patterns in large databases. DM extracts the information of interest and is also able to find new key elements that might influence the outcome, thus increasing the amount of data that can be utilized [13, 21]. Finding new trends that influence success rates for IVF are important for the patient and the clinician to have realistic outcome expectations. To help clinicians predict pregnancy success rates, they created a hybrid intelligence model that used DM to integrate genetic algorithm-based and decision tree learning techniques that extracted information from the IVF patient records. They found that this model not only helped predict outcomes but suggested modified IVF treatment according to individual patient characteristics. A downside of the study is that the model they created used data from only one IVF center. However, if the centers united to share information, their pooled data could significantly be expanded to represent a wider population with increased accuracy. Other studies suggest using ANN systems to predict IVF outcomes by using a learning vector quantizer which allows generalization and standard parameters for enhanced predictive power [20]. Another aspect of IVF and AI is the possibility of identifying the most viable oocytes and embryos. An AI system used by Manna et al. suggested combining AI to extract texture descriptors from an image (local binary pattern) and assembling it by using an ANN [22]. These results proved to be above average when compared to current methods and could help to select the best possible oocytes or embryos noninvasively and objectively. Furthermore, they highlight the advantages of this technology in selecting the most viable embryos, even in countries where legislation precludes embryo selection by sex.

Cancer screening

Neural network models are being used to deliver prognoses in patients with ovarian cancer. Ovarian cancer is a catchall for heterogeneous neoplasm, and there is a great variation in histology and inpatient presentation, such as existing tumor stages. In a report done by Enshaei et al., their results demonstrated that ANN was able to predict survival with a 97% accuracy [23]. The AI systems they developed have the potential of providing an accurate prognosis. Similarly, Norwitz et al. have created an AI software that can predict prognosis in patients with ovarian cancer more precisely than current methods [18, 23]. It can also predict the most effective treatment according to the diagnosis of each patient. Long-term survival rates for advanced ovarian cancer are poor; thus, more targeted therapies are needed. 

Researchers at Brigham and Women’s Hospital and Dana-Farber Cancer Institute have been using AI to manipulate large amounts of micro ribonucleic acid (RNA) data to develop models that can potentially diagnose early ovarian cancer [23]. Currently, no screening for ovarian cancer exists despite it being a common gynecological cancer. Thus, most cases are diagnosed in advanced stages, leading to a high five-year mortality rate. The AI neural network was able to keep up with the complex interactions between micro RNA and accurately identified almost 100% of abnormalities that represented ovarian cancer, as opposed to an ultrasound screening test that was able to identify abnormal results less than 5% of the time. This non-invasive testing consists of measuring micro RNAs from a serum sample, which can be paramount for the future management of ovarian cancer. 

To identify patients at risk of more aggressive tumors, a newly developed AI system has been created to scan ovarian cancer cells; this system can help identify irregularly-shaped nuclei that correlate with tumor aggressiveness [19, 23]. Scanning by an AI system can be incorporated in routine biopsies to identify these risk factors related to DNA instability and choose therapies accordingly. Evasion of the immune system has been identified in the misshapen nuclei in aggressive ovarian cells, indicating that there can be a response to immune-targeted treatments, such as onco-immunotherapy.

AI has recently been incorporated into oncology through commercial applications that use the algorithm to match patient data with current clinical trials nationwide and respective investigational drugs per patient [24-26]. IBM's Watson for Oncology uses AI in conjunction with patient data to help guide cancer management, which has proven efficient for breast cancer patients [27].

Furthermore, AI has outperformed human experts in interpreting cervical pre-cancer images [26]. The current screening consists of visual inspection of the specimen collected during a Papanicolaou (PAP) smear and using acetic acid to visualize whitening in the tissue which would be indicative of disease. Despite its convenience and low cost, it lacks accuracy. AI deep learning algorithms can gather a large number of images related to cervical cancer screening and appropriately identify diseased tissue. It also ensures that the test is convenient, accurate, and cost-effective, unlike the current method. Minimal training is required, and the results are immediate; thus, patients can receive treatment in the same visit [24-26]. 

Gynecological surgery

In surgery, the application of physical AI has been utilized more than virtual AI. Virtual AI uses established patient factors, repetitive patterns, and treatment algorithms to predict the outcome, as opposed to the surgical field, which has many independent variables. Some of these variables are the consistency of different tissues, the skills of each surgeon, the changes that are done in the surgical field while operating, and unique differences between patients and their pathology; ultimately, these unique factors make it challenging to create an algorithm [27].

Areas in which AI has assisted gynecological surgery include those related to imaging and spatial awareness. AI can aid the surgeon by providing better imaging before and during surgery. The creation of three-dimensional printing (3DP) that replicates the surgical site is far superior to its two-dimensional (2D) counterpart, as it represents a more precise version of the actual model [28-29]. This allows a more accurate preoperative plan, hence diminishing errors in the operating room. 3DP can also provide different materials that can resemble the tissues that would be encountered, thus providing realistic practice for trainees and unprecedented preoperative planning [29]. Ultimately, a 3DP image helps to navigate the surgical field and increase awareness of the area involved, thereby protecting its surrounding structures. This has proved useful in one case report for deep infiltrating endometriosis (DIE) [27, 29]. A 3DP model was derived from preoperative magnetic resonance imaging (MRI) and then later compared retrospectively with the surgical findings. The surgeons agreed the 3DP image assisted in planning to calculate the depth, extent, and involvement of the adjacent structures and how to proceed accordingly. These models can potentially serve as adjuncts for preoperative planning where depth and spatial relationships are challenging to analyze by the standard 2D image alone. 

AI has also helped decrease operative time and accuracy, which subsequently decreases operative complications [27]. This has been done via the utilization of augmented reality. Augmented reality consists of a computer that can reconstruct objects taken from the real-world and enhance them virtually to create a more informative visual image [30-31]. One review of augmented reality in surgery summarizes some of the shortcomings of this technology, such as increased cost, concern for a latency of the system, and that the head-mounted display is heavy and impractical for long surgeries [32]. Furthermore, augmented reality might cause nausea and vomiting due to “simulator sickness,” which would be less than ideal during surgery. Augmented reality might also demonstrate too much information and increase clutter, thus distracting the surgeon from the task on hand, and would be most helpful in an immobile structure so that mobile organs (like the uterus) are not ideal for this technology. Despite certain disadvantages, the authors of the review concluded that, overall, there are positive benefits to augmented reality, such as increased precision, safety, and a time reduction in performing procedures [32]. Nonetheless, further studies and improvements still need to be done to take full advantage of its potential benefits [31].

Other examples of computer systems assisting in surgery are evident in robotics and computer-assisted platforms. With the help of these platforms, the possibility to opt for a minimally invasive route during surgery becomes feasible and can help reduce invasive surgeries, their associated risks, and reduce operative times. Some robotics can even decrease tremors to promote accuracy [27]. Additionally, the introduction of instruments, such as 3D laparoscopic surgery which aids in-depth visibility, has the potential to provide better surgical outcomes [32].

AI has aided in enhancing spatial awareness, cautioning surgeons if certain vessels or structures of importance are concealed, identifying them in a timely matter, and being able to protect vital structures. An example is isolating the ureters during gynecological surgery. A study was done using an endoscope system based on AI using algorithms to estimate the depth and location of the ureters, which proved to enhance accuracy and safety [33].

Discussion

Machine learning can significantly improve healthcare; however, the downsides of machine AI need to be considered. Ethical dilemmas, such as the potential of human biases when creating computer algorithms, need to be addressed [34]. Health care predictions can vary by race, genetics, and gender amongst other variations, and failure to take these into account might over or underestimate patient risk factors. As stated in the review by Ho et al. concerning AI analytics in healthcare, it will become the responsibility of the clinician to ensure that AI algorithms are developed and applied appropriately [35]. It is imperative that healthcare continues to operate by ethically defined guidelines to sustain trustworthiness and that medicine continues to prioritize the good of the patient. AI continues to be promising since it can decrease healthcare costs and reduce clinician workload as it collaborates in decision-making. 

AI will become more intertwined in clinical practice, and there are machine learning instruments that no longer require the revision of a clinician to interpret their results, such as in the case of the IDX-DR AI device (IDx Technologies Inc., Coralville, IA), which detects mild diabetic retinopathy [36]. Since this software does not require interpretation by a specialist, more primary care physicians can use it in their practice, potentially decreasing the workload for specialists, and making the interpretation available for all types of clinicians. This can potentially be applied to obstetrics regarding the interpretation of FHR and CTG interpretations. 

There are instances where AI-based computer-aided design (CAD) has led to decreased diagnostic precision in the interpretation of mammography [37]. AI alone was shown to be superior to a single radiologist in detecting breast cancer. However, in practice, an individual radiologist reviews these images, and due to bias, might dismiss CAD suggestions [35]. Nonetheless, when two specialists review the image, it is most likely to undergo additional testing if the readers disagree with each other. Thus, in this study, it was unclear if AI was cost-effective when compared to interpretation by two radiologists. Additional disadvantages are that AI-based CAD cannot explain the reasoning behind a decision. Thus, in case of a misinterpretation by the software, it is difficult to determine if the manufacturer or the radiologist that interpreted the data is at fault. Creating agencies that can develop standards that validate and ensure product quality and accuracy need to be established. Additionally, the algorithms created need to deliver under a broad range of settings that can adequately mirror real-world conditions to which they are being applied clinically [38]. Real-world settings are easier to mimic using a larger amount of data which can be obtained by accessing patient records. However, patient confidentiality becomes a challenge when retrieving personal information. The development of blockchain systems can potentially help to keep patient information confidential. This would allow the simultaneous sharing of data between centers, incorporate it into the AI software, and allow it to continue expanding its array of records which would lead to improved accuracy [39].

Professionally, clinicians need to familiarize themselves with AI in order to revise it so that the machine can provide accurate information. Furthermore, it needs to be flexible in adopting new information so the machine needs to continue learning and changing accordingly. Moreover, the data must be representative of the population being evaluated in a realistic clinical setting. Despite challenges to AI, it has the overall potential of revolutionizing patient care by providing a more accurate diagnosis, alleviating the burden of work for clinicians, decreasing healthcare costs, and providing a baseline analysis in tests where substantial differences in interpretation between specialists exist. Further developments in medical AI will continue. Clinicians must embrace them, yet be wary, and when necessary, recognize its advantages and drawbacks to continue providing the best patient care. 

## Conclusions

We have discussed the different forms of AI, prior research on AI, its advantages and disadvantages, potential challenges, and the future of AI application in OB/GYN. Our comprehensive review shows that AI has a promising future in overcoming diagnostic challenges and improving treatment modalities and patient outcomes in OB/GYN. Further studies need to be done to decrease bias when creating algorithms and to increase adaptability in the system, enabling the incorporation of new medical knowledge as new technology surfaces. Practitioners must also take safety measures to ensure that the analysis is valid and accurate. AI is not meant to replace practitioners but rather to serve as an adjunct in decision-making. Ethically, the use of patient records might bridge patient confidentiality since large amounts of data are required to enable AI systems to have access to the large and varied population statistics which are encountered in clinical settings, hence providing realistic and accurate predictions.
